# Recent advances in enzymatic synthesis of β-glucan and cellulose

**DOI:** 10.1016/j.carres.2021.108411

**Published:** 2021-10

**Authors:** Gregory S. Bulmer, Peterson de Andrade, Robert A. Field, Jolanda M. van Munster

**Affiliations:** a Department of Chemistry and Manchester Institute of Biotechnology, The University of Manchester, 131 Princess Street, Manchester M1 7DN, UK; b Scotland's Rural College, Edinburgh, UK

**Keywords:** Glucans, Cellulose, Glycosyltransferases, Glycoside hydrolases, Glycoside phosphorylases, Nanostructures

## Abstract

Bottom-up synthesis of β-glucans such as callose, fungal β-(1,3)(1,6)-glucan and cellulose, can create the defined compounds that are needed to perform fundamental studies on glucan properties and develop applications. With the importance of β-glucans and cellulose in high-profile fields such as nutrition, renewables-based biotechnology and materials science, the enzymatic synthesis of such relevant carbohydrates and their derivatives has attracted much attention. Here we review recent developments in enzymatic synthesis of β-glucans and cellulose, with a focus on progress made over the last five years. We cover the different types of biocatalysts employed, their incorporation in cascades, the exploitation of enzyme promiscuity and their engineering, and reaction conditions affecting the production as well as in situ self-assembly of (non)functionalised glucans. The recent achievements in the application of glycosyl transferases and β-1,4- and β-1,3-glucan phosphorylases demonstrate the high potential and versatility of these biocatalysts in glucan synthesis in both industrial and academic contexts.

## Highlights

• Carbohydrate-active enzymes are vital for the bottom-up preparation of β-glucans.
• Glycosyltransferases and glycosyl hydrolases widened the scope for β-glucan synthesis.
• Glycoside phosphorylases can produce various well-defined cellulose-like materials.
• Recent applications demonstrate their high potential, robustness and versatility.

## Graphical abstract

**Figure:**
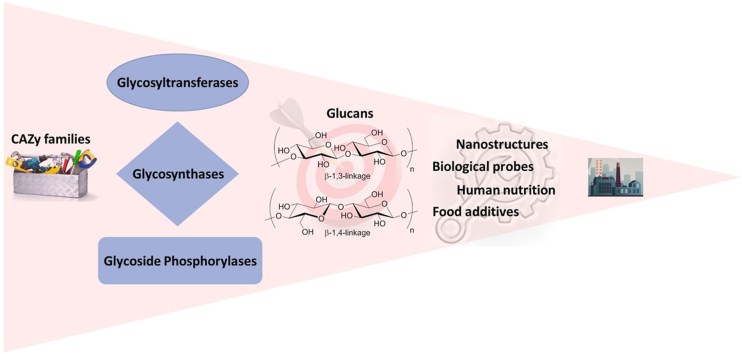

## 1 Introduction

### 1.1 β-glucan structure

**Fig. 1:**
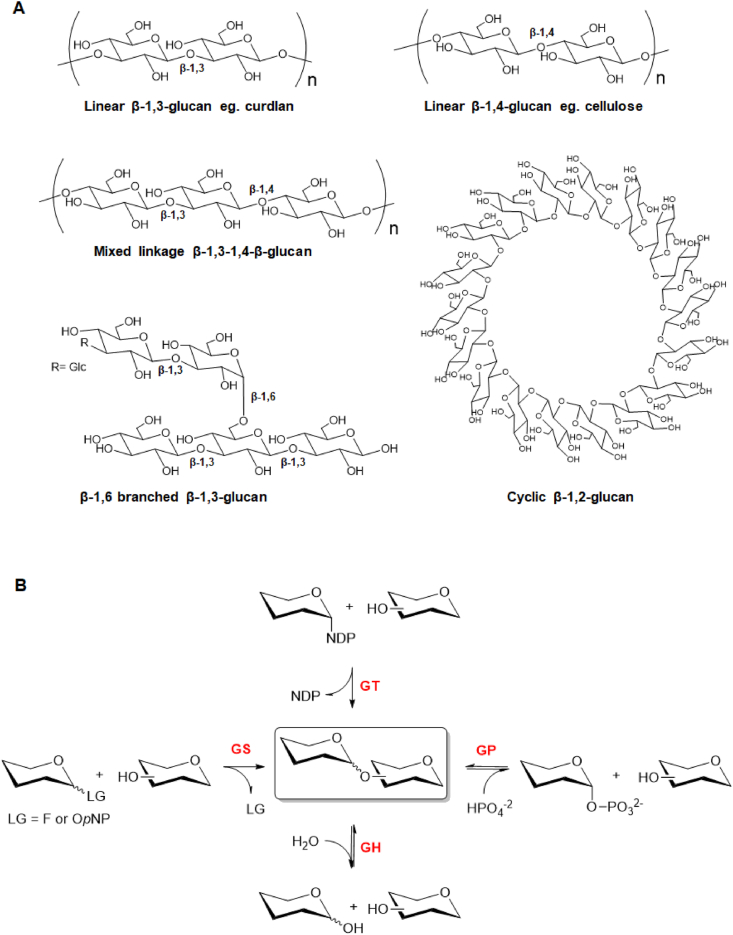
**A)** structure of natural glucans. **B)** Glycosidic bond formation and/or cleavage catalysed by different CAZy families. **GS** = glycosynthases (Leaving Group: Fluorine or O-*para*-nitrophenyl); **GT** = glycosyltransferases (NDP: nucleotide diphosphate); **GP** = glycoside phosphorylases and **GH** = glycoside hydrolases. (Part **B** was adapted from Ref. [23]).

β-glucans (Fig. 1A) are major structural components of plants and fungi that are of significant importance to a large range of fields such as nutrition, microbial pathogenicity and the renewable fuel sector. β-glucans, including the major biopolymer cellulose, circumscribe a range of large, natural glucose oligosaccharides linked by β-glycosidic bonds of varying linkage types and as a group comprise some of the most abundant carbon sources on Earth. The type and proportion of linkage types within β-glucans has an impact on both structure and behavior, with different forms of β-glucans occurring depending on their source [1]. The enzymatic production of these glucans by glycosyl transferases, glycosynthases and glycoside phosphorylases is a rapidly changing field, of which the recent advances will be discussed in detail.

#### 1.1.1 β-1,4 glucan

The β-1,4 glucan cellulose is the most abundant biopolymer on Earth with 2 × 10^11^ tons produced annually [2]. Consequently, this renewable source is of great interest in the production of bioethanol, other high-value products and the overall success of a future bio-based economy [3]. Cellulose is held together by van der Waals forces and an extensive network of intra/inter chain hydrogen bonds that yield microfibrils with highly ordered regions (crystalline), thus resulting in structural stability and robustness [4]. Wood and cotton fibres are the most common sources of cellulose [5], and the polysaccharide is also produced by bacteria [6]. The production of nanocellulose (cellulose nanocrystals - CNC and cellulose nanofibers - CNF), based on its isolation from cellulosic biomass, is considered a top-down method due to high energy consumption and harsh chemical conditions (strongly acidic and basic) [7]. Nanocellulose has become a valuable class of material due its inherent characteristics and potential for a wide range of advanced industrial applications (composites, electronics, sensors, cosmetics, pharmaceutical, biomedical, etc) [[8], [9], [10]].

#### 1.1.2 β-1,2 glucan

Cyclic β-1,2-glucans (CβG) are found in a limited number of symbiotic and pathogenic gram negative bacteria, including the genera *Agrobacterium*, *Rhizobium* and *Brucella* [11]. Within these genera CβGs enable the invasion of, and survival within, host cells. CβGs are non-cytotoxic, can activate mammalian dendritic cells and enhance antigen-specific CD4^+^/CD8^+^ T cell responses, thus offering potential as an adjuvant in antimicrobial therapies [12]. In bacteria CβGs are known to sequester iron in order to protect against iron-induced toxicity [13]. With a backbone of around 17–25 glucose units, these glucans reside within the periplasmic space and are responsible for osmotic homeostasis on the cell wall [12].

#### 1.1.3 β-1,3 glucan

Cereal β-glucans found in oats, barley and rye have a combination of β-1,4 and β-1,3 linkages and form long-chain linear polysaccharides of high molecular weight, with the β-1,3 linkages causing an irregular coiled structure. Fungal β-glucans are β-1,3 linked polysaccharides with side chains of varying length attached to the backbone through β-1,6-linkages [14]. They are a major constituent of the fungal cell wall that can form up to 40% of the dry wall mass [15]. As seen in the Plantae, fungal glucan composition varies by species, with mushrooms normally containing short β-1,6-linked branches whilst the yeasts display longer β-1,6 side branches [16]. Such glucans are a major component of dietary fibre and have food stabilizing properties. Furthermore β-1,3-glucans have been shown to hold anti-tumor activities [17] in addition to their use as β-glucan micro-particles for the delivery of therapeutics [18]. Functioning as key immunomodulatory agents, they stimulate the immune system and induce cellular immunity [19]. Linear β-1,3 glucan polysaccharides such as curdlan (bacterial), callose (plant) and paramylon (microalgal) have high-molecular weight and are water-insoluble [20]. These polysaccharides are stabilised by a broad network of intra/inter chain hydrogen bonds that can exist in helical or random coil structures [21,22]. Their potential application in several industrial sectors ranges from farming, nutrition, cosmetics to therapeutics. For instance, curdlan has been used as gelling agent in foods, and there has been also interest in using β-1,3 glucans as functional dietary fibres and immunomodulatory agents [24,25]. In fact, the regulatory effects of dietary β-glucans (food supplements or part of daily diets) have been recently reviewed to highlight a potential role in cancer control due to their ability to modulate a variety of biological responses [26].

### 1.2 Current need for enzymatic synthesis of β-glucans

Oligo- and polysaccharides have conventionally been produced with low yields from natural sources by hydrolysis and used for biological assessment mainly as heterogeneous mixtures. Inefficient processes for extraction and purification have contributed to high-cost production that limits the access to pure and structurally well-defined short/long linear β-glucans [21,24]. Chemical synthesis of glycans, despite all the progress in the field [27], remains challenging due to the need of specific building blocks as reactants, solubility challenges and poor regio-/stereoselective reactions that yield heterogenous materials. In this respect, enzymatic synthesis has naturally become an attractive approach to enable the bottom-up preparation of oligo- and polysaccharides in a regio- and stereocontrolled manner, thus resulting in high synthetic precision and site-specific modifications [23,28,29].

Enzymes offer a variety of benefits in comparison to chemical catalysts, specifically their ability to operate under mild conditions thus allowing for greater process efficiency and enabling more sustainable routes to chemicals [30,31]. Briefly, ideal biocatalysts constitute the following properties in respect to catalysis: high activity; high regio-/stereo-selectivity; lack of unwanted side activities such as donor or product hydrolysis; stability both when stored and within reactions. Additionally, simple, high-yield heterologous expression within popular expression hosts such as *Escherichia coli* is desirable, as is promiscuity towards a variety of modified substrates to produce a diverse repertoire of natural and non-natural products [32]. Attainment of this suite of traits can be enabled by enzyme discovery or engineering to improve activity, scope and yield. Examples of such progress in the production of β-glucans and cellulose have been demonstrated across a variety of glycoenzymes, recent examples of which will be covered in this review.

## 2 Glycosyltransferases

### 2.1 Glycosyltransferase activity

Glycosyltransferases (GTs) (Fig. 1B) are found widely across nature and are responsible for the synthesis of glycosidic linkages between a non-activated acceptor carbohydrate and (usually) an activated sugar donor [33]. This activity can occur onto a variety of different biomolecules such as mono-, di- and oligosaccharides [34,35], LPS [36] and peptidoglycan [37]. GTs that utilise activated nucleotide sugars (carbohydrates linked to a nucleoside mono- or diphosphate e.g. CMP, UDP, GDP or TDP) are termed Leloir glycosyltransferases. Non-Leloir GTs utilise non-nucleotide donors such as activated phosphorylated sugars (phosphorylases, discussed in more detail in section 3) or non-activated donors in the form of sucrose or starch-derived oligosaccharides (transglycosidases) [35,38]. Leloir GTs are responsible for the synthesis of the majority of carbohydrates in nature and as such are of great interest (and one of the first enzymatic tools considered) for those wanting to enzymatically synthesise commercially or medically important glycans. Glycosyltransferases can be promiscuous in both acceptor and donor substrate acceptance, and thus provide biocatalysts with potentially exploitable side reactions with rates that in many cases have been demonstrated to be sufficient for exploitation in glycoside synthesis [32]. Generally, GTs display high glycosyl donor and acceptor selectivity resulting in products with high regio- and stereo-selectivity and have frequently been used in preparative scale, one-pot single/multi-enzyme glycosylation cascades to synthesise a variety of natural products as recently reviewed in Refs. [33,38].

### 2.2 Glycosyl donor recycling

Although commercially available, the activated glycosyl donor monosaccharides required by Leloir GTs are relatively expensive, thus presenting a problem in chemoenzymatic reactions whereby larger quantities of product are required. To rectify this issue there has been a concerted effort in the in situ recycling of nucleotide sugar donors. UDP-glucose can be produced via activity of sucrose synthase (SuSy), whereby sucrose is hydrolysed into fructose and UPD-glucose when in the presence of UDP. SuSy has been utilised in a variety of biocatalytic processes as reviewed here [39]. Mutants of bacterial SuSys have enabled the development of more efficient variants allowing improved UDP-Glc generation. Furthermore, analysis of the plant SuSys, such as *Glycine max* (soybean), has revealed members more active than their bacterial counterparts [40]. The lower activities of bacterial SuSys have been attributed to residues in the nucleoside binding site having higher affinities for ADP, rather than UDP. Introduction of plant residues at these sites resulted in up to 60-fold decreases in K_m_ for UDP, producing enzymes more suitable for industrial applications [41]. In GT-dependent biocatalysis, achieving the typical efficiency targets required for industrial processes is often problematic. Therefore, the ~100 g scale production of UDP sugars such as UDP-Glc is vital for commercially viable biotransformations. Bioreactor cultivation of *E. coli* expressing *Acidithiobacillus caldus* SuSy was shown to produce 100 g_product_/L and operate a space-time yield of 10 g/L/h. UDP-Glc could then be purified using chromatography-free downstream processing with yield of 86% [42].

### 2.3 Glycosyltransferase mediated glucan synthesis

#### 2.3.1 β-1,4 glucan synthesis

**Fig. 2:**
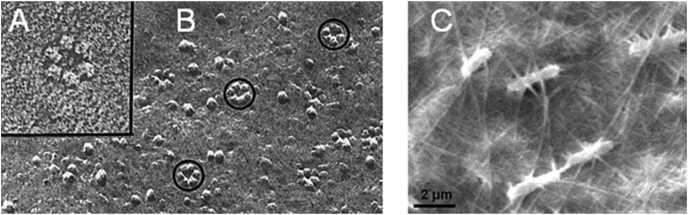
**A** & **B** Freeze fracture replica of CSC (inset) within the plasma membrane of *Zinnia elegans*, adapted from Ref. [45]. **C** SEM of *K. rhaeticus* surrounded by bacterial cellulose, adapted from Ref. [46].

The in vitro biosynthetic synthesis of (1,4)‐β‐d‐glucan has proved a challenging task that for many years was unattainable [43]. Within plants, the predominant producers of cellulose, the natural biosynthetic machinery consists of a complex membrane-embedded multi component system called the cellulose synthase complex (CSC) (Fig. 2A and B) [44]. This machinery has proven to be extremely difficult to express and maintain in a functional form. While CSC activity from a variety of plant cell extracts had initially been demonstrated [[47], [48], [49]], only recently CSCs have been functionally reconstituted in vitro [50]. Heterologous expression of *Populus tremula x tremuloides* CesA8 (*Ptt*CesA8) enabled the in vitro production of cellulose and its subsequent processing into cellulose microfibrils. This polymerisation was dependent on a lipid bilayer environment (in addition to Mn^2+^ ion presence), as solubilisation of these lipid vesicles by detergent removed this activity. Whether activity is abolished due to enzymatic sensitivity to the detergents or alteration of the enzyme quaternary structure is unknown [50].

Reconstitution of such bacterial cellulose synthases (Bcs), which bridge the periplasm through the use of subunits [51], has also been achieved. Purified cellulose synthase BcsA and membrane-anchored, periplasmic subunit BcsB from *Rhodobacter sphaeroides* were utilised in vitro for the production of cellulose of DP 200–300, whereby BcsB inclusion was essential for the catalytic activity of BscA. Unlike *Ptt*CesA8, BcsA remains active in a variety of non-denaturing detergents and is independent of lipid-linked reactants, but requires the allosteric activator cyclic-di-GMP [52]. The tailoring of reaction conditions for bacterial cellulose synthases can drive cellulose morphology in vitro. Detergent extracted CSC's from *Komagataeibacter xylinus* demonstrated how varying temperature and centrifuging reactions, to mimic an anisotropic environment, can alter the product profile. Optimal production of cellulose, with a II crystal structure and average DP of 60–80, occurred at 35 °C without centrifugation. Lower temperatures (e.g. 13 °C) resulted in lower yield, likely due to reduced particle movement. At higher temperatures (42 °C) protein denaturation and aggregation affected cellulose morphology. Increasing rates of centrifugation resulted in decreased cellulose production and a reduction in reducing end detection by BCA assay [53].

The application of synthetic biology has enabled the effective tuning of bacterial cellulose production, as demonstrated in the genetic manipulation of *Komagataeibacter rhaeticus* (Fig. 2C). Development of such a genetic toolkit allowed engineering of inducible cellulose production when required, thus improving yields by avoiding the emergence of cellulose-nonproducing mutants that can arise when cellulose is synthesised constitutively. Gene expression of *K. rhaeticus* can be induced within the cellulose pellicle and offers a route to the controlled production of new cellulose-based, spatially and temporally patterned biomaterials. Post hoc addition of *E. coli* expressed proteins with a fused CBD (Cellulose Binding Domain) to cellulose produced functionalised materials and was implemented in the production of cellulose-based garments containing GFP, demonstrating feasibility at macroscale [46].

The synthesis of Glc-β-1,4-Glc linkages from nucleotide sugars by glycosyltransferases is predominantly limited to the use of plant and bacterial cellulose synthases. However, promiscuity in donor and acceptor specificity of other glycosyl transferases has been exploited. Bovine β4GalT1 has been demonstrated to transfer Glc from UDP-Glc onto GlcNAc and Glc-terminated acceptors at low efficiencies, producing β-1,4 linkages [32]. More recently it has been demonstrated that *Neisseria meningitidis* glycosyltransferase, LgtB, can be used for the in vitro polymerisation of glucose from UDP-glucose via the generation of β-1,4-glycosidic linkages, producing oligosaccharides from DP 2–10. Furthermore LgtB is permissible to biocatalytic cascades and the production of glucose oligosaccharides with tailored functionalities that can be utilised to inform on the activity of various plant cell wall degrading enzymes [54].

#### 2.3.2 β-1,3 glucan synthesis

The study of β-1,3 glucan synthesis in vivo has identified the responsible glycosyltransferases, which are also commonly referred to as glucan synthases (GLS) [14]. Various groups have demonstrated how membrane bound GLS’ could be extracted from fungal and plant cells for use in in vitro assays, e.g. via the preparation of microsomal membranes and subsequent detergent fractionation [[55], [56], [57]]. Recently, through inhibition of cellulose synthesis, the stress response formation of β-1,3 callose can be induced with cultured protoplasts of *Nicotiana tabacum* (tobacco) and *Arabidopsis thaliana.* Fibres of different morphologies being produced depending on the plant species, e.g. hollow with average width of 12 μm or densely packed with a width of 8 μm respectively [58]*.* Detergent-isolated *Betula* protoplasts containing callose synthase were shown to secrete β-1,3 callose bundles in vitro when incubated with a high concentration of Ca^2+^ ions. The lengths of these fibres were considerably longer as those from the two herbaceous plants [59]. These GTs require membrane protein solubilisation (through the use of detergents such as CHAPS) for use in in vitro reactions which, in tandem with their large nature and poor stability in solution, means there are few examples of their successful use in in vitro reactions.

## 3 Glycoside hydrolases

### 3.1 Glycoside hydrolase

Glycoside hydrolases (GHs) catalyse the breaking of glycosidic bonds but many GHs are also capable of functioning in the reverse direction through either the displacement of equilibrium (thermodynamic control) or through the use of activated sugar donors that intercept the reactive glycosyl-enzyme intermediate via kinetically controlled transglycosylation [[60], [61], [62]]. These methods offer opportunities to leverage the large natural repertoire of GH specificities, to enable routes for the enzymatic synthesis of designer glycans.

A variety of enzymes with natural transglycosylation abilities are reported in the literature and therefore of interest when making building blocks for the production of derivatised oligo- and polysaccharides. One such example is a β-glucosidase from *Penicillium funiculosum*. The recombinantly expressed rBgl6 demonstrated the transglycosylation of glucose to form the β-1,6 linked glucose dimer gentiobiose with a yield of 23% [63]. The bacterial endo-1,3-β-d-glucanase from *Formosa algae* is able to perform transglycosylation of the acceptors methyl-β-d-xylopyranoside, methyl-α-d-glucopyranoside, 4-methylumbelliferyl-β-d-glucoside and glycerol, and utilizes laminarin as the donor. Of these acceptors glycerol was the most efficient whereby the most predominant species in the reaction mix were the transglycosylation products with a DP of 2–12 [64]. Other exoglucanases, such as ExgD, carry out transglycosylation using various primary alcohols to produce alkyl-glycosides. Using methanol, ethanol and propanol as acceptors and *p*-nitrophenyl cellobioside as the glycosyl donor, methyl-, ethyl- and propyl-cellobiosides were synthesised with yields of 87%, 55% and 30% respectively – offering enzymatic routes to non-ionic surfactants normally produced by acid catalysis [65].

### 3.2 Glycosynthases

#### 3.2.1 Glycosynthase activity

The basis for glycosynthase research began in 1991 with the first non-natural, biosynthetic, in vitro route to cellulose that was performed using a wild-type retaining *Trichoderma viride* cellulase whereby β-d-cellobiosyl fluoride was utilised as the substrate in a acetonitrile/acetate buffer mix (required to promote transglycosylation) [66]. However, the use of wild type glycosidases has significant drawbacks, as the polymerisation products can act as a substrate for the enzyme, resulting in hydrolysis of the product – vastly reducing potential yields [67]. To alleviate this issue, in 1998, Mackenzie et al. developed the first glycosynthase (Fig. 1B); a genetically engineered exo-glycosidase capable of synthesising oligosaccharides through utilisation of an activated glycosyl donor in an anomeric configuration opposite to the natural substrate. The glycosynthase is unable to hydrolyze reaction products due to mutation of the catalytic residue, resulting in an inability to form the requisite R-glycosyl-enzyme intermediate. Mutation of endo-glycosidase, to enable the use of oligosaccharides of different degree of polymerizations to act as glycosyl donors, has also been reported [68]. Besides glucosynthases, a broad range of synthases accepting other glycans has been developed, as recently reviewed elsewhere [69].

#### 3.2.2 β-1,2 glycosynthases

Various phenolic compounds are known to have positive impacts on human health, however such compounds often have poor bioavailability which can be improved with glycosylation. Protein engineering enabled the production of the β-1,2 transglycosylating BGL-1-E521G that was able to utilise phenolic acceptors such as epigallocatechin gallate (EGCC) in addition to pNP-sugars, whilst using α-glucosyl-fluoride as the donor. Consequently a variety of β-1,2-glucosylated phenolic compounds were produced in a regioselective manner [70].

#### 3.2.3 β-1,3 glycosynthases

Glycosynthases have been successfully employed to generate β-1,3-glucan for example as demonstrated for *Hordeum vulgare* E231G mediated self-condensation of α-laminaribiosyl fluoride and 3-thio-α-laminaribiosyl fluoride to polymers of > DP 20 and DP 6–8 respectively [71]. Synthesis of mixed-linked 1,3-1,4 β-glucans from di-, tri- and tetrasaccharide donors has also been achieved, whereby tuning of the β-1,3 and β-1,4 linkage ratio produced glucans that do not occur in nature, thus demonstrating the potential to modify glucan structures through enzymatic synthesis [72]. Recently progress has been made towards the development of β-1,3-glucan synthases that dispense with the need for fluorinated donors.

The glycosyl fluoride donors commonly required by glycosynthases are often unstable at high temperatures, meaning reactions are limited to temperatures of 30–37 °C which is suboptimal for many thermophilic enzymes of interest. Consequently the development of in situ-generated α-glycosyl formate donors, in an example of chemical rescue, allowed the use of both the fluoride donor or a exogeneous formate nucleophile to produce a β-1,3 disaccharide [73]. Such an approach offers an attractive alternative to the use of expensive activated donors and was further utilised in the development of a thermotolerant β-glycosynthase (*Tn*Bgl3B) from a *Thermotoga neapolitana* β-glucosidase. In addition to using a fluoride donor, rational design of the glycosynthase also allowed the use of the formate “non-classical’’ system (without a glycosyl fluoride donor) and enabled reaction temperatures of 80 °C permissible for the thermotolerant enzyme. Using pNp-Glc as both donor and acceptor the *Tn*Bgl3B variant consequently produced pNP-laminaribioside (pNP-Glc-1,3-Glc), after longer incubations pNP-cellobioside was also produced [74]. The recent application of a glycosynthase immobilised and used in flow for the synthesis of speciality glycans suggests an emerging field for the design and implementation of glycosynthases in flow chemistry and could be key to improving their commercial viability [75].

#### 3.2.4 β-1,4 glycosynthases

Mutation of an endoglucanase from *Humicola insolens* (producing *Hi*Cel7B E197A) enabled the polymerisation of cellobiosyl fluorides for the production of regioselectively modified oligo- and polysaccharides. Both unsubstituted and modified mono- and disaccharide acceptors were utilised to create a variety of natural and novel β-1,4 linked compounds. *Hi*Cel7B was able to accept C-6 position functionalised α-cellobiosyl fluorides containing various groups including bromine and thioglucosyl groups and represented the first self-condensation of a cellobiosyl fluoride donor to produce cellulose [76]. *Hi*Cel7B E197A was further exploited to produce modified cellulose derivatives through polymerisation of 6′-azido-α-cellobiosyl fluoride to produce alternating 6-azido-6-deoxycellulose. Such alternating 6-azido-6-deoxycelluloses can be acetylated then subsequently reduced to amines. Conversely, alternating 6-azido-6-deoxycelluloses can undergo copper(I)-catalysed azide-alkyne cycloaddition (CuAAc - click chemistry) with Alexa Fluor 488 Alkyne to form conjugated alternating 6-azido-6-deoxycellulose [77].

The need for high-throughput technologies in enzyme discovery and development has driven the creation of assays. The development of colorimetric assays for glycosynthase screening allows the improved discovery and selection in a high-throughput manner [69] whilst the screening of diverse, synthetic gene libraries allows a systematic approach to glycosynthase discovery [78].

## 4 Glycoside phosphorylases

Glycoside phosphorylases (GPs) naturally catalyse the cleavage of glycosidic bonds in the presence of inorganic phosphate, releasing sugar 1-phosphate and shorter glycans (Fig. 1B). Since these phosphorolysis reactions are reversible, due to equivalent free energy released by the anomeric bond cleavage in both directions, these enzymes are effectively used to catalyse the synthesis of a wide range of glycosides in a regio- and stereospecific manner. These carbohydrate-active enzymes (CAZy) are capable of producing disaccharides or oligosaccharides with a broad range of glycosidic linkages (except β-1,1 and α/β-1,6) from several acceptors (D-Glc, D-GlcNAc, maltose, trehalose, sucrose, nigerose, etc) and mostly α-d-glucose 1-phosphate (α-D-Glc-1P) as donor substrate. GPs have been classified into glycoside hydrolase (GH) and glycosyltransferase (GT) families according to their sequence similarity (CAZy database; http://www.cazy.org/). Most GPs (EC 2.4.1.x) belong to GH families and are grouped into retaining and inverting phosphorylases, [23]. Some good reviews have covered in more detail phosphorylases classification [23], structure and mechanism [79,80], and use as catalysts for glycoside synthesis [23,29,81]. Herein, we provide a brief overview on recent use of GPs to synthesise β-1,3 and β-1,4 glucans focussing on the following enzymes: cellodextrin and cellobiose phosphorylases, as well as Pro_7066/laminaridextrin and laminaribiose phosphorylases.

### 4.1 β-1,4-D-Glucan phosphorylases (cellodextrin and cellobiose phosphorylases)

#### 4.1.1 Cellulose-like materials (long-chain insoluble oligosaccharides)

Characterised from various anaerobic bacteria and obtained in recombinant form in *E. coli* under standard conditions, cellodextrin phosphorylase (CDP, EC 2.4.1.49) is a homodimer that belongs to the GH94 family and is the most studied phosphorylase polymerising β-1,4-glucan. CDP is an inverting enzyme that produces cellodextrins (cello-oligosaccharides, oligocelluloses or cellulose oligomers) from α-D-Glc-1P and D-Glc/short β-1,4-glucans as natural donor and acceptor substrates, respectively [82]. Different degree of polymerisation (DP) towards long-chain insoluble (DP ≥ 7) or short-chain soluble (DP ≤ 6) oligosaccharides can be achieved depending on the reaction conditions. Although optimum pH and temperature are dependent on the enzyme source, CDPs are very stable under a wide range of pH (5.0–8.6) and temperature (37–60 °C), as well as in mixture of aqueous buffer and organic solvents. Equally important, the nature of the substrates has a significant impact upon structural features, such as crystallinity and morphology. CDP's great potential as biocatalysts for synthetic applications relies on the fact that non-natural substrates can be reasonably tolerated, thus giving rise to tailor-made cellulose-like materials with different nanostructures.

**Fig. 3:**
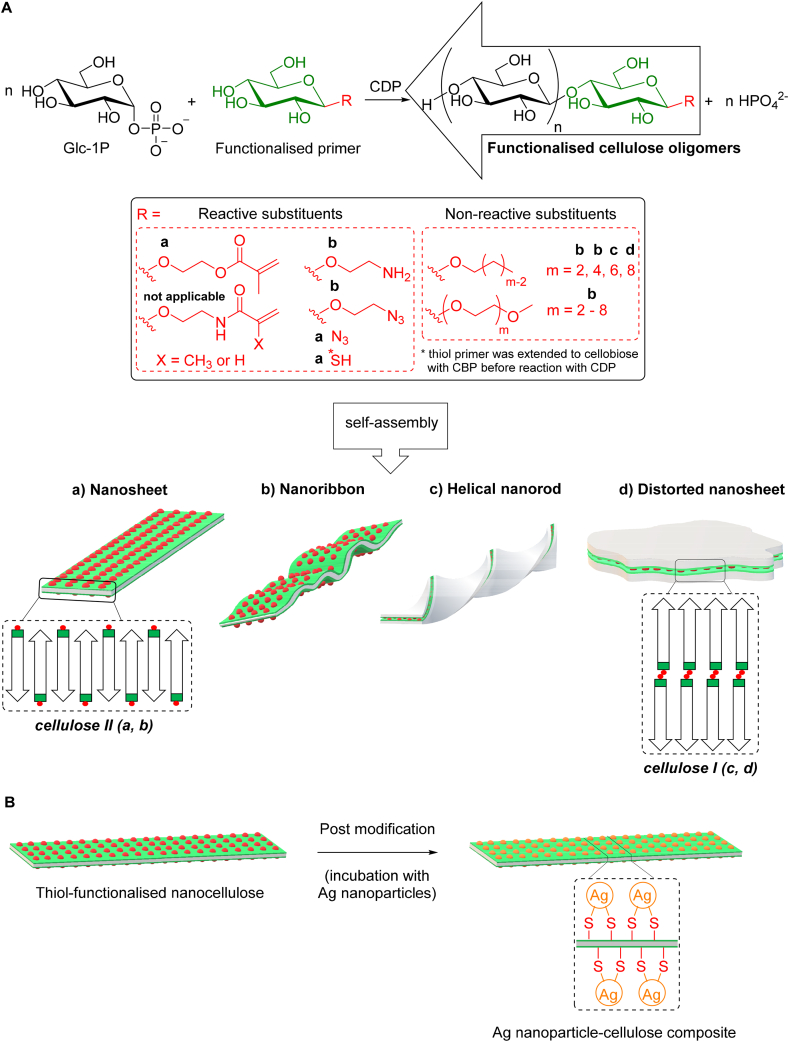
**A**) Insoluble cellulose oligomers self-assembled into ordered nanostructures produced by cellodextrin phosphorylase (CDP)-catalysed synthesis from functionalised acceptors with reactive or non-reactive substituents at the anomeric position. **B**) Post modification example using thiol-functionalised cellulose oligomers in the presence of silver nanoparticles to produce nanocellulose composite (adapted from Ref. [87]).

**Fig. 4:**
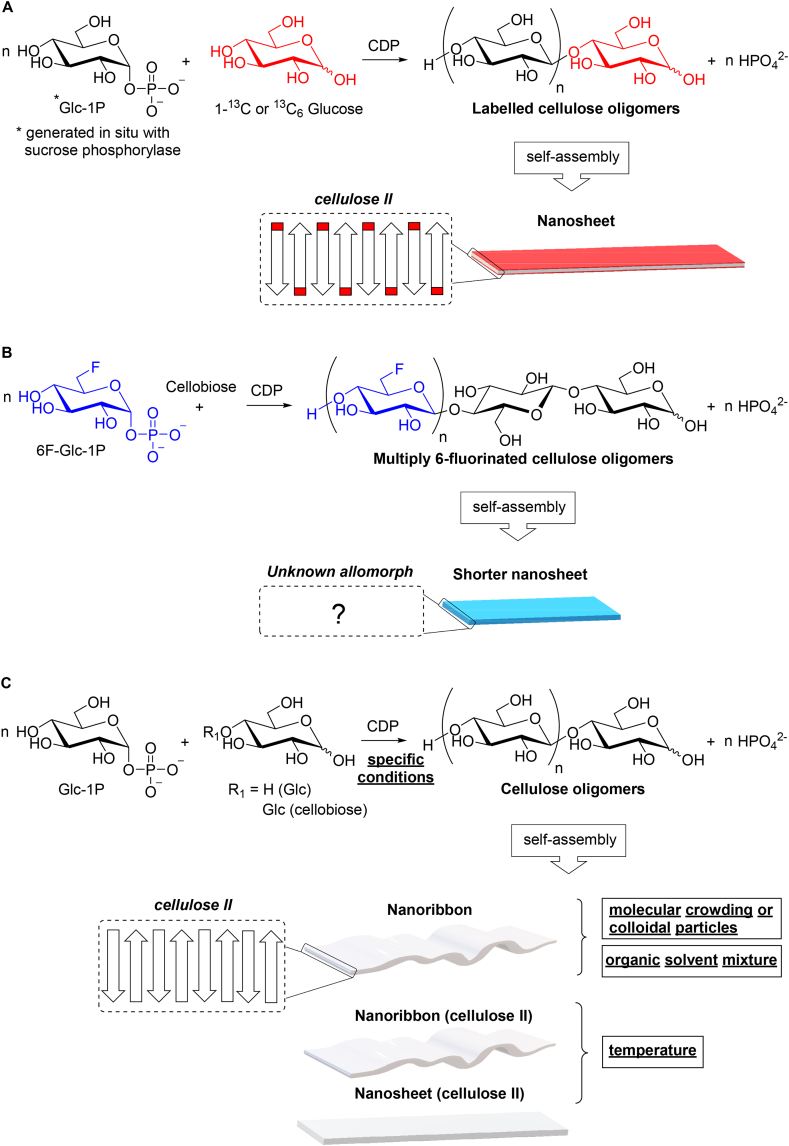
Insoluble cellulose oligomers self-assembled into ordered nanostructures produced by cellodextrin phosphorylase (CDP)-catalysed synthesis from selectively labelled acceptors (**A**), donor bearing site-specific probe (**B**), and non-functionalised substrates under specific reaction conditions (**C**).

A comparison of the acceptor substrate specificity of CDP from different sources has shown its permissiveness towards a broad range of natural and functionalised d-glucosyl at the reducing end, whereas the same promiscuity is not observed to its donor substrate. For instance, d-cellobiose and longer cello-oligosaccharides are considerably better acceptors than plain D-Glc, which yields very poor activity (0.5 to < 8%) [28]. The ability of CDP to better accommodate longer (DP ≥ 2) and modified acceptor substrates is consistent with the structural data (PDB code 5NZ8). CDP has an open active site with a more accessible acceptor binding pocket due to the disposition of the two subunits within the dimer interface and the significant rearrangement of the catalytic and opposing loops that delineate the active site [82]. In order to harness its poor substrate specificity, most recent works have been focussed on studying self-assembly, crystallinity, morphology and potential properties of nanostructures produced from iterative glycosylation of β-d-glucose acceptors (primers) functionalised at the anomeric position. The Serizawa group has pioneered the use β-d-glucose derivatives with anomeric substituents as substrates for CDP (*Clostridium thermocellum*) (Fig. 3A). Reactive substituents have been exploited to provide additional reactivity of the cellulose oligomers for post modification with other molecules, whereas non-reactive substituents have been tested as a means to control the self-assembly processes. Furthermore, in situ self-assembly using only plain substrates have been investigated under different reaction conditions (Fig. 4C).

##### 4.1.1.1 Functionalised acceptors with reactive substituents

In a proof-of-concept work, crystalline cellulose oligomers with surface-reactive azide groups, synthesised from α-D-Glc-1P and β-d-glucose 1-azide by CDP under aqueous and mild conditions, were postmodified with 1-ethynyl pyrene in DMF via click chemistry to produce a sheet-like 2D crystalline cellulose II (ca. 5 nm thickness) with broad fluorescence emission due to the pyrene units partially forming excimers on the nanomaterial surface. This result highlights the potential use of CDP to generate well-defined cellulose-like materials bearing spectroscopic reporter groups incorporated in a site-specific manner [83]. In a similar approach involving surface-reactive substituents, highly crystalline 2D vinyl-cellulose nanosheets (ca. 6 nm thickness) were synthesised via enzymatic polymerisation of α-D-Glc-1P from the primer 2-(glucosyloxy)ethyl methacrylate and postmodified by covalent incorporation at different loadings into poly(ethylene glycol) (PEG) matrix through thiol-ene Michael addition to generate a series of nanocomposite hydrogels with enhanced mechanical strength [7]. Later, the Loos group extended the repertoire of vinyl-based oligocelluloses catalysed by CDP using five anomerically pure β-d-glucosyl primers elegantly synthesised from D-Glc/cellobiose and hydroxy-alkyl (meth)acrylates or (meth)acrylamides with commercial β-glucosidases. Although not focussing on post modification, the authors also suggested the potential use of these structures for further (co)polymerisation with different monomers in the context of property-tunable hydrogels [84]. In another study, Serizawa et al. investigated the protein adsorption properties of surface-aminated cellulose oligomers synthesised from α-D-Glc-1P and 2-aminoethyl β-D-glucoside. The resulting crystalline nanoribbons (ca. 5 nm thickness) were tested as solid support for specific proteins (BSA, cytochrome *c*, fibrinogen, IgG, lysozyme, transferrin, and trypsin) and revealed that only those negatively charged at pH 7.4 (fibrinogen, IgG and transferrin) were adsorbed onto the nanoribbons due to effective interaction with the primary amine on their surface. The aminated nanoribbons also showed no cytotoxicity against mammalian cells, thus demonstrating potential biomedical applications [85]. Most recently, functionalised cellulose oligomers synthesised from α-D-Glc-1P and 2-azidoethyl β-D-glucoside by CDP were used to prepare functionalised cellulose paper. The paper was efficiently modified through heterogenous nucleation-based self-assembly (impregnation) of the oligomers bearing terminal azide dissolved in aqueous alkaline solution and subsequent neutralisation. Post modification of the fibre surface of cellulose paper was simply performed via click chemistry with alkynylated biotin for further high-sensitivity detection of IgG in a proof-of-concept diagnostic application. The successful noncovalent functionalisation and post modification of paper together with its long-term functional stability may boost the development of sustainable devices [86]. Similarly, the Nidetzky group used reducing end thiol-modified nanocellulose, synthesised from α-D-Glc-1P and 1-thio-β-d-glucose via linear cascade with cellobiose phosphorylase (CBP, EC 2.4.1.20) and CDP, as a template for the directed assembly of silver nanoparticles in order to produce functional cellulose nanocomposite (Fig. 3B). The resulting thiol-containing nanosheets of crystalline cellulose II (ca. 5 nm thickness) with high loading silver nanoparticles, selectively bound and well dispersed on the surface, were assessed against *Escherichia coli* and *Staphylococcus aureus* and showed excellent antibacterial activity. This study may expand the scope of the bottom-up approach towards the synthesis of functionalised nanocellulose with respect to metal nanoparticle composites [87].

##### 4.1.1.2 Functionalised acceptors with non-reactive substituents

Since the poor colloidal stability of enzymatically synthesised nanocelluloses in aqueous solution restricts the formation of highly ordered structures, Serizawa et al. also studied the impact of β-d-glucosyl primers bearing hydrophilic [88] and hydrophobic [89] non-reactive substituents on self-assembly (Fig. 3A). Oligo(ethylene glycol) (OEG) was initially selected due to its highly hydrated and dynamic nature in aqueous phases that could result in stability. Interestingly, the enzymatic reaction produced cellulose hydrogels consisting of crystalline nanoribbon networks with different structural features based on the OEG chain lengths. The cellulose DP shifted from 9 to 11 and the thickness of the nanoribbons increased from 5 to 6 nm with increase in the chain, which in turn decreased the width from several hundred to below 100 nm. These results suggested that the OEG chains modulated the self-assembling process of the cellulose oligomers possibly by colloidal stability of the nanoribbon precursors [88]. In a follow-up work, alkylated cellulose oligomers synthesised from alkyl β-D-glucoside primers varying in chain lengths (2, 4, 6, 8 carbons) self-assembled into different nanostructures such as nanoribbons, helical nanorods and distorted nanosheets. Surprisingly, the alkyl chain length affected not only the morphology but also the crystallinity of the material, probably by effective modulation of the intermolecular interactions of cellulose oligomers thus resulting in active assembly controlling. Shorter alkyl chains (2 and 4 carbons) yielded hydrogels composed of long nanoribbons corresponding to cellulose II allomorph, whereas longer chains (6 and 8) generated dispersions composed of helical nanorods or distorted nanosheets resembling cellulose I allomorph [89].

##### 4.1.1.3 Modified acceptors/donor with labelled atoms or probes

Cellulose oligomers containing selectively labelled atoms or site-specifically incorporated probes are important to report on local structure and environment, thus enabling their detailed characterisation and better understanding for broader application in advanced nanomaterials. Solid-state NMR analyses of cellodextrins corresponding to cellulose II nanosheets, enzymatically synthesised from ^13^C-enriched D-Glc acceptors and α-D-Glc-1P (Fig. 4A), revealed that the reducing-end units located on the surface tend to have β-anomeric configuration, which would be more sterically stable than alpha [90]. More recently, the Field group investigated the impact of single and multiple site-specific incorporation of fluorine into cellodextrin chains prepared from modified acceptors (2F-, 3F- and 6F-D-Glc) and α-D-Glc-1P with CBP as well as from modified donor (6F-α-D-Glc-1P) and d-cellobiose with CDP, respectively. As expected, the presence of a single fluorine at the reducing end of each chain had no substantial impact upon morphology and crystallinity as evidenced by the typical cellulose II precipitated nanosheets with average DP ca. 8. Conversely, the presence of multiple 6-deoxy-6-fluoro-d-glucose units yielded shorter nanosheets of unprecedented crystalline allomorph (Fig. 4B) with higher average DP (ca. 10) and longer chains (up to DP 15). Furthermore, advanced solid-state NMR methods enabled deciphering of the water-exposed and interior chemical environments for different carbon sites [91].

##### 4.1.1.4 Non-functionalised substrates

The highly ordered hierarchical formation of nanostructures through in situ self-assembly of enzymatically synthesised insoluble cellulose oligomers is not only affected by chemical functionalisation, but also by different reaction conditions using natural substrates (Fig. 4C). Inspired by the concept of molecular crowding, which occurs in intracellular environments with high concentration of different macromolecules and has considerable impact on the dynamics of biomolecular self-assembly [92], the Serizawa group synthesised cellulose oligomers from α-D-Glc-1P and D-Glc in the presence of concentrated water-soluble polymers (dextran, poly(*N*-vinylpyrrolidone) - PVP, PEG and Ficoll) [93,94] to promote self-assembly of the cello-oligosaccharides. The reactions under in vitro macromolecular crowding resulted in stable hydrogels composed of well-grown crystalline nanoribbon networks of cellulose II, regardless of the polymer species, whereas the conventional rectangular sheet-like precipitates were formed in the absence of the polymers. It has been suggested that the aggregation of particles was possibly suppressed by increased solution viscosity and consequent decrease in diffusion rates of colloids, thus leading to dispersion stability for further growth into long nanoribbons networks to form a hydrogel. This concept was applied to produce a composite hydrogel via in situ self-assembly and cross linking of enzymatically synthesised cellulose oligomers in gelatin solution at 60 °C followed by cooling [93]. In a follow-up study, an equally robust double-network hydrogel was prepared by pH-triggered self-assembly of cellulose oligomers with gelatin. Insoluble cello-oligosaccharides (ca. DP 10) dissolved in alkaline solution (1 N NaOH) self-assembled upon pH decrease (7.4) in the presence of a warm acidic solution of gelatin to yield a hydrogel with improved stiffness, which was assumed to be based on entanglement between the networks of gelatin and the nanoribbon-shaped fibres of cellulose oligomers [95]. The pH neutralisation approach was also studied in the presence of a more complex mixture, such as serum-containing cell culture media, resulting in a physically cross-linked hydrogel of cellulose nanoribbon networks with anti-biofouling properties. The cells grew into spheroids (cell aggregates) and with no unfavourable sedimentation as the self-assembly progressed in a controlled manner for 3D cell culture, thus suggesting an attractive prospect towards biocompatible soft materials [96]. Considering that coexisting colloidal particles can also induce increase in viscosity and reduce stabilisation of colloidal dispersions, it has been hypothesised that colloidal particles could be used as crowding reagents towards nanoribbon network formation. In this sense, mechanically stable composite hydrogel with colloidal particles spatially immobilised (trapped) within cellulose nanoribbon network was produced by enzymatic synthesis and self-assembly of the oligomers in the presence of rod-like cellulose nanocrystals as model colloidal particles. The hybrid hydrogel proved to be organic solvent-resistant and its stiffness was dependent on the concentration of the nanocrystals, suggesting physical cross-linking to the nanoribbons [97].

Besides the molecular crowding and colloidal particles approaches, the Serizawa group also investigated the influence of temperature [98] and organic solvents [99] upon in situ self-assembly of cello-oligosaccharides synthesised from α-D-Glc-1P and D-Glc (Fig. 4C). Precipitated nanosheets were formed at higher temperatures (60 - 40 °C), whereas nanoribbon networks and dispersed nanosheets were produced by lowering the temperature to 30 °C and 20 °C, respectively. With this temperature shift, a decrease of DP from 10 to 8, and increase in crystallinity (from 52% to 66%) and similar dispersity index were observed. The temperature-directed assembly resulting into different cellulose II nanostructures was possibly driven by the synergy between reduced hydrophobic effect and concomitant induced self-crowding effect [98]. While rectangular precipitated nanosheets are traditionally obtained in aqueous buffer, reactions in mixture with organic solvents (DMSO, DMF, acetonitrile or ethanol) can yield hydrogels of well-grown nanoribbon networks. The solvent control over oligomerisation-induced self-assembly was rationalised through mechanistic studies based on the Kamlet-Taft parameters, which showed that the nanoribbon formation was only triggered by solvents with high β-values (hydrogen bond acceptor ability - DMSO and ethanol). This data suggests that the solvation resulting from hydrogen bonding between the organic solvent and the cello-oligosaccharides prevented aggregation and consequent precipitation towards nanosheet formation, thus allowing a higher-order structure to be formed. It was also noticed that cellulose II with similar average DP (8–10) was obtained in all reactions. However, the crystallinity was higher in the presence of organic solvent (ca. 64% vs 52%) due to higher uniformity of the chain lengths, evidenced by the lower dispersity index (ca. 1.2 vs 1.9) [99].

A simple approach to produce hydrogel consisting of nanoribbon networks (cellulose II with average DP 7–8 and ca. 4.5 nm thickness) in aqueous buffer was performed using d-cellobiose [100,101] instead of conventional D-Glc as acceptor, which typically gives rise to precipitated nanosheets [102,103]. In this case, the distinct morphological outcome under the same reaction conditions was attributed to the effect of short-chain soluble oligosaccharides present in the supernatant of d-cellobiose reaction due to kinetic control. Soluble oligomers might accumulate once their rates of formation and self-assembly seemed to be similar. Conversely, no soluble oligomers were detected in the glucose reaction as the rate of cellobiose formation (rate-limiting step) was probably slower than the self-assembly rate [100,102]. In a follow-up study, higher concentration of D-Glc (200 and 500 mM) also resulted in nanoribbon networks hydrogel since the soluble oligomers possibly worked as dispersion stabilisers of the precursor particles, thus suppressing precipitation/aggregation and favouring growth [104]. It is worth mentioning that previous use of d-cellobiose [105] resulted only in precipitated nanosheets because the reaction conditions were significantly different (lower concentration of reactants and enzyme). This emphasises the impact of setting suitable conditions in the process of generating tailor-made nanocellulosic materials.

#### 4.1.2 Ingredients for animal and human nutrition (short-chain soluble oligosaccharides)

**Fig. 5:**
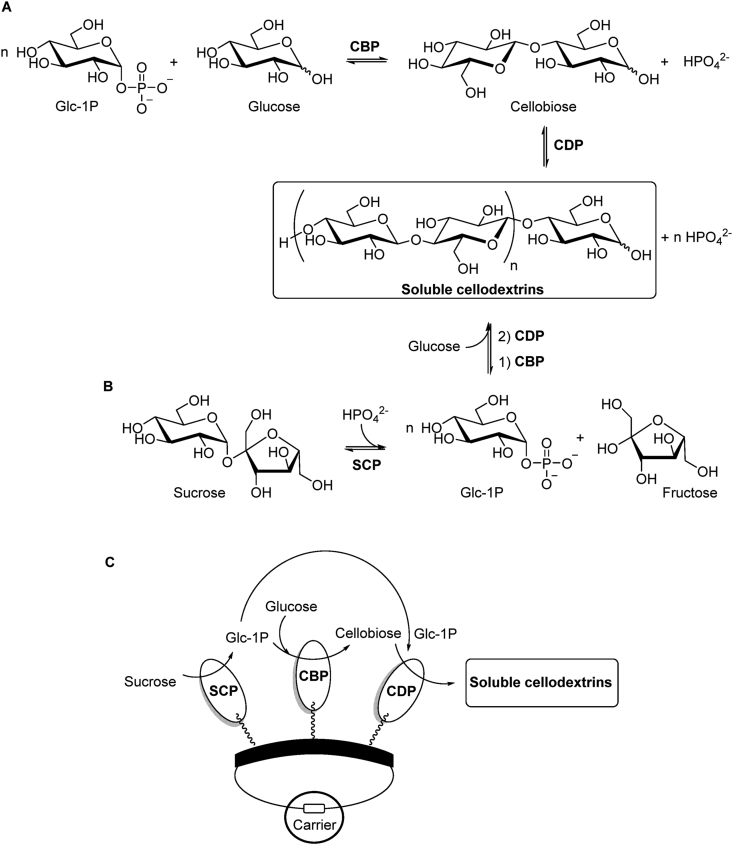
Controlled biocatalytic synthesis of soluble cellodextrins (DP ≤ 6, n = 1–4) using cellobiose phosphorylase (CBP) and cellodextrin phosphorylase (CDP) in a linear cascade reaction from α-D-Glc-1P and D-Glc (**A**), and via a three-enzyme phosphorylase cascade in solution (**B**) or on solid support (**C**) from sucrose and D-Glc including sucrose phosphorylase (SCP). (Part **C** was adapted from Ref. [111]).

The state-of-the-art application of CDP also involves the synthesis of short-chain soluble oligosaccharides (DP ≤ 6) as means to access structurally defined model substrates as biological probes, focussing on the potential prebiotic effect for farm animals [106] and dietary fibres for humans [107]. Initially, a typical mg scale synthesis of short oligosaccharides has been studied by Loos et al. using α-D-Glc-1P and d-cellobiose in a constant molar ratio (20:1). The results showed a tendency towards the production of cellodextrins with reduced chain length in the presence of higher concentration of the acceptor substrate [108]. In this regard, Nidetzky et al. proposed a controlled biocatalytic synthesis of soluble cellodextrins using CBP (*Cellulomonas uda*) and CDP (*Clostridium cellulosi*) in a linear cascade reaction from α-D-Glc-1P and D-Glc (Fig. 5A), which is economically more attractive and 7-fold more soluble than d-cellobiose [109]. The optimised conversion of these substrates into soluble products with DP ranging from 3 to 6 (ca. 96 wt%) was mainly controlled by an appropriate molar ratio of α-D-Glc-1P/D-Glc (4:1) and in situ precipitation of released inorganic phosphate with Mg^2+^ (MgCl_2_) to shift the equilibrium towards product formation. The activity ratio between both enzymes also proved to be an important parameter, showing best results with excess of CBP (3 U/mL) over CDP (2 U/mL) producing a final product concentration of 36 g/L within 2 h with a conversion yield of 98% based on glucose. Although the disaccharide is a better substrate than glucose (ca. 50-fold), the two-enzyme reaction outcome is very similar in terms of product distribution and concentration when compared to the single step CDP conversion of d-cellobiose under the same conditions.

Recently, Nidetzky et al. also studied the integrated production of soluble oligomers via a three-enzyme phosphorylase cascade [110]. This biocatalytic process was developed to convert expedient substrates, such as sucrose and glucose, into short-chain cellodextrins using sucrose phosphorylase - SCP (*Bifidobacterium adolescentis*), CBP and CDP (Fig. 5B). The iterative β-1,4-glycosylation of D-Glc from α-D-Glc-1P, generated in situ from sucrose and inorganic phosphate, was successfully achieved after optimisation of key parameters. The balance of the three phosphorylase activities (10:3:2 U/mL) along with the ratio sucrose/glucose, inorganic phosphate concentration and reaction time were crucial to DP control and increased concentration of soluble cellodextrins with DP 3–6 (40 g/L). Additionally, a convenient and efficient two-step procedure for product isolation was accomplished with high purity (≥95%) and yield (ca. 92%) via selective microbial conversion of the undesirable sugars (sucrose, fructose and glucose) using *S. cerevisiae*, followed by organic solvent precipitation. In a follow-up work, they exploited the cascade synthesis of soluble cello-oligosaccharides upon co-immobilisation of the three phosphorylases on solid support [111] (Fig. 5C). The chimeric enzymes harbouring the binding module were immobilised via ionic interaction on the same carrier to harness the effect of spatial proximity. As observed for the cascade reaction in solution, the phosphorylases on solid support also required a defined ratio of activities for an efficient DP-controlled synthesis. Despite finding the optimal activity ratio and a good loading, the catalytic effectiveness was just above 50% and the molar yield concentration of soluble products was low (56%, 25 g/L) compared to the high sucrose conversion (90%). This was ascribed to the insoluble product formation (DP ≥ 8), which seems to occur at a higher rate when compared to the phosphorylases in solution. In this case, the reaction time was shortened to produce only the desired products but it also reduced their molar yield concentration to ca. 30% (12 g/L). The recyclability assessment revealed that these enzymes retained 85% of the overall initial activity after five cycles, though substantial release of CBP from the carrier was observed. Although promising, these results clearly show the challenges and limitations posed by a complex biocatalytic cascade performed on solid support.

Envisioning the scale up synthesis of such interesting soluble cello-oligosaccharides with potential industrial application, the Nidetzky group optimised their enzymatic production and assessed their growth stimulation among probiotic bacteria [112]. The one-pot biotransformation from sucrose and glucose using the three phosphorylases in solution was carried out in gram scale and optimised for a maximum soluble oligomers concentration of 93 g/L, which represents a 2.4-fold improvement compared to previous results [110]. The insoluble product formation was also reduced below 10 mol% after adjusting the enzyme activity ratio from 10:3:2 to 20:6:2 U/mL. The soluble products (DP 3–6) showed substantial growth stimulation (up to 4.1-fold with respect to the maximal cell density) for some probiotic strains when compared to known oligosaccharide prebiotics (inulin and *trans*-galacto-oligosaccharides) and d-cellobiose. Thus, these results support the importance of soluble cellulose oligomers as selective functional carbohydrates with considerable prebiotic potential.

**Fig. 6:**
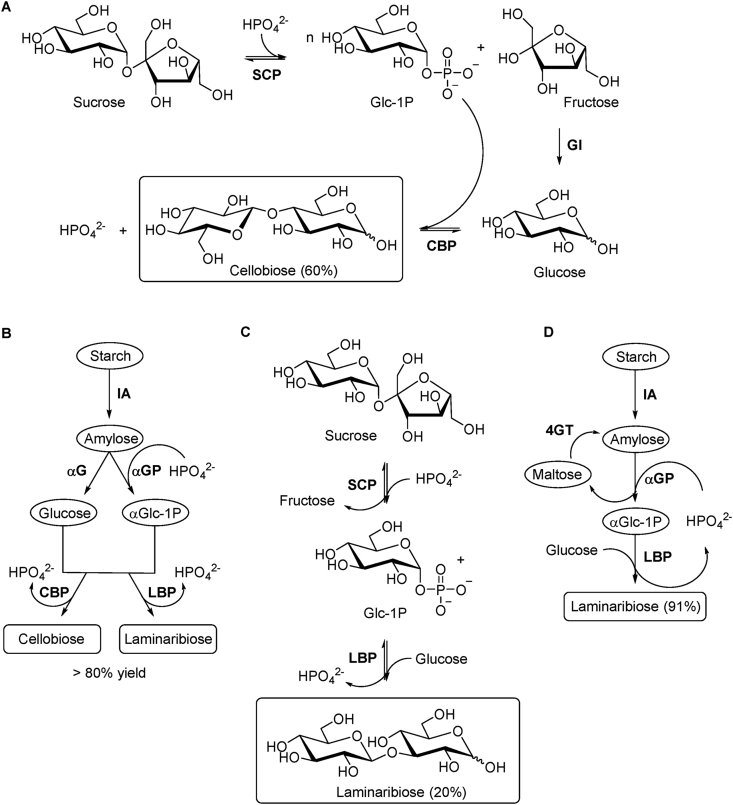
**A**) One-pot synthesis of d-cellobiose from sucrose catalysed by sucrose phosphorylase (SCP), glucose isomerase (GI) and cellobiose phosphorylase (CBP). **B**) Enzymatic platform for the synthesis of various disaccharides from starch employing isoamylase (IA), α-glucosidase (αG) and α-glucan phosphorylase (αGP) in parallel, and a disaccharide phosphorylase such as CBP or laminaribiose phosphorylase (LBP). **C**) d-laminaribiose synthesis from sucrose and D-Glc in one-pot reaction containing individually immobilised SCP and *E. gracilis* extract with enriched LBP activity. **D**) Cascade synthesis of d-laminaribiose from starch and glucose using IA, αGP, LBP and 4-α-glucanotransferase (4GT), which was used to recycle maltose into malto-oligosaccharides for continuous production of α-D-Glc-1P.

Amongst the soluble cellodextrins, cellotriose is thought to be the most potent prebiotic. For instance, it is the preferred substrate for *Bifidobacterium breve*, a major probiotic bacterium in the human intestine [113]. Since the efficient synthesis of defined short-chain cello-oligosaccharides by wild type phosphorylases remains challenging, a recent study evaluated engineered CDP (*Clostridium cellulosi*) and CBP (*Cellulomonas uda*) as possible biocatalysts for the enriched synthesis of cellotriose from α-D-Glc-1P and d-cellobiose [114]. Despite several site directed-mutations to disrupt a specific region of the CDP active site to hamper further chain elongation, all mutants were still capable of elongating the chains towards higher DPs. A more promising result was achieved upon a directed mutation to optimise a well-known mutant (CBP OCP2) [115], which has five mutations located in the catalytic cleft that confer some degree of activity and affinity for d-cellobiose. The improved mutant (OCP2_M52R) showed 4-fold higher activity and affinity for d-cellobiose as well as reduction in further elongation. Consequently, cellotriose was synthesised with the highest purity (82%) and yield (73%) to date, and only trace amounts of cellotetraose and cellopentaose were observed. Interestingly, glucose can be also used as an acceptor for cellotriose synthesis despite the new mutant also exhibiting higher affinity for the disaccharide. Therefore, this study highlights a positive perspective in engineering CBP for the defined production of cello-oligosaccharides longer than d-cellobiose.

d-cellobiose has been also a valuable and commercially attractive sugar with respect to zero-calorie sweeteners and as potential food additive. Although its synthesis from sucrose by SCP and CBP has been under implementation for industrial production [116], current research focussing on atom economy has been exploited via multi-enzyme one-pot biotransformation approaches with mathematical model assistance to predict optimal reaction conditions and enzyme loading ratio [117,118]. In this sense, d-cellobiose was synthesised from sucrose by three thermophilic enzymes with 60% yield within 10 h. The process involved phosphorolysis of sucrose by SCP (*Thermoanaerobacterium thermosaccharolyticum*) to form α-D-Glc-1P and fructose, which was isomerised by glucose isomerase (*Streptomyces murinus*) into D-Glc for further disaccharide synthesis by CBP (*Clostridium thermocellum*) (Fig. 6A) [117]. More recently, a cost-effective platform for the in vitro synthesis of disaccharides from starch was successfully developed employing four enzymes. Starch was debranched with isoamylase to produce amylose for subsequent parallel conversion into D-Glc and α-D-Glc-1P with α-glucosidase and α-glucan phosphorylase, respectively, followed by final catalysis with CBP (*Clostridium thermocellum*) (Fig. 6B). Amongst other valuable disaccharides synthesised in this study (laminaribiose, trehalose and sophorose), d-cellobiose was produced within 8 h with yield higher than 80% from low and high concentrations of starch. Since in vitro multi-enzymatic cascades have emerged as a promising biomanufacturing platform, this process proved to be robust and could represent an alternative approach to the current disaccharide production [118]. Although not covered in this review, manno-oligosaccharides are also of interest in the production of nutraceuticals and pharmaceuticals due to their prebiotic effects and many other biological properties [119]. In this regard, it is important to mention the relevance of β-1,4-D-mannan phosphorylase (GH130) as a cost-effective biocatalyst towards the in vitro synthesis of pure linear short β-1,4-mannan chains (DP 16) [120], thus emphasising its potential for biotechnological applications.

### 4.2 β-1,2-D-Glucan phosphorylases

In the presence of inorganic phosphate >100 g scale β-1,2-glucan synthesis was achieved enzymatically using a two-enzyme system of sucrose phosphorylase from *Bifidobacterium longum* subsp. *longum* and 1,2-β-oligoglucan phosphorylase from *Listeria innocua* expressed in *E. coli* [121]*.* A similar scale was also achieved utilising sophorose as the acceptor [122]. Few tools currently exist to elucidate the nature of proteins that interact with CβGs and consequently this difficulty is reflected in the literature, however advances are being made in the form of acid depolymerised oligosaccharide microarrays and derived probes to follow such interactions [123].

### 4.3 β-1,3-D-Glucan phosphorylases (laminaridextrin and laminaribiose phosphorylases)

The families GH94 and GH112 have been previously reported to contain inverting GPs with regio- and stereospecificity towards the synthesis of β-1,3-linked disaccharide or oligosaccharides. The in vitro preparation of disaccharide has been catalysed by bacterial laminaribiose phosphorylase (LBP, EC 2.4.1.31) [124,125] whereas crystalline oligosaccharides (DP 30) have been produced by a partially purified β-1,3-D-glucan phosphorylase extract from the microalga *Euglena gracilis* [126]. In order to understand the glycobiology of *E. gracilis* and circumvent the laborious preparation of extract as source of catalyst, the Field group identified the algal β-1,3-glucan phosphorylase and uncovered a new family of phosphorylases (GH149). The candidate phosphorylase sequence (EgP1) was selected through proteomic analysis from cellular protein lysate and expressed in *E. coli* for in vitro characterisation and confirmation of its catalytic activity. Moreover, EgP1 orthologous sequences led to a bacterial metagenomic sequence that was expressed in *E. coli* (Pro_7066) and assessed to validate its function as β-1,3-D-glucan phosphorylase. Despite comparable activity in the synthesis of short oligosaccharides (DP up to 12) from D-Glc and α-D-Glc-1P, kinetic data suggested EgP1 preference for glucose and cellobiose whereas Pro_7066 showed similar catalytic efficiency for all tested acceptors (DP1 to DP6) [127]. In a continuous effort to identify and characterise new β-1,3-D-glucan phosphorylases, a phosphorylase sequence from heterokont algae *Ochromonas* spp (OcP1) was identified along with bacterial orthologs, thus leading to the identification of a new GH family (GH161). The activity of GH161 family members was established from a bacterial GH161 gene sequence (*papP*, *Paenibacillus polymyxa*) cloned and expressed in *E. coli*. Recombinant PapP showed neither synthetic activity in the presence of D-Glc and α-D-Glc-1P, nor phosphorolysis activity towards Glc-Glc disaccharides with different linkages. Nonetheless, phosphorolysis assays with DP3 to DP6 revealed its preference for longer substrates. These results strongly suggest that PapP can only operate on β-1,3 linear oligosaccharide acceptors with DP ≥ 2, evidencing its different specificity from GH149 glucan phosphorylases [128]. These studies expand the repertoire of GPs acting on β-1,3-D-glucans and provide information on substrate preference/specificity.

In order to gain understanding of β-1,3-D-glucan phosphorylases at the molecular level and elucidate the basis for oligosaccharides chain length specificity of GH149 enzymes, the X-ray crystallographic structures of Pro_7066 in the absence of substrate (PDB code 6HQ6) and in complex with laminarihexaose (PDB code 6HQ8) were solved. Although the overall domain organisation is similar to GH94, Pro_7066 enzyme contains two additional distinct domains flanking its catalytic region and a surface oligosaccharide binding site where laminarihexaose was accommodated, which is distant from the catalytic site and may be involved in the recognition of longer substrates [129]. The crystallographic structure of the GH94 laminaribiose phosphorylase from *Paenibacillus* sp YM-1 (*Ps*LBP, PDB code 6GH2) demonstrated its specificity for disaccharides. Interestingly, *Ps*LBP was not only limited to act on d-laminaribiose as previously described [124], but also catalysed the synthesis of β-d-mannopyranosyl-1,3-d-glucopyranose from D-Glc and α-D-Man-1P, albeit with reduced catalytic efficiency (ca. 150-fold). Structural data of *Ps*LBP in complex with α-D-Glc-1P and α-D-Man-1P, together with saturation transfer difference (STD) NMR studies, revealed a similar binding mode for α-D-Man-1P due to its close overlapping with α-D-Glc-1P. However, it was observed the loss of an important hydrogen bond between the axial hydroxy group at C2 and a key residue in the active site, thus possibly contributing to the low reaction turnover [130]. This study pioneered the molecular detailed recognition of an unnatural donor substrate by GPs as a means to provide background knowledge to harness their prospective as biocatalyst for synthetic applications. More recently, wild-type Pro_7066 and CDP showed reasonable tolerance to a variety of sugar 1- phosphates in a multi-milligram-scale synthesis of fragments of human milk oligosaccharides (HMOs) [131], which are currently a hot topic in enzymatic syntheses [132]. Kinetic data of these enzymes indicated general low efficiency (<1%) for the unnatural donors α-D-Gal-1P, α-D-GlcN-1P and α-D-Man-1P compared to α-D-Glc-1P in the presence of both d-laminaribiose and d-cellobiose, though the transference of glucose onto both acceptors was effective. By producing mixed new β-1,4- and β-1,3-linkages from different donor substrates, the idea of ‘one enzyme to one linkage’ can no longer be applied to these phosphorylases in spite of their stereo- and regiospecific. Despite the natural tendency to generate long oligosaccharides, single turnover also was observed in some reactions. Considering that the lack of suitable enzymes hampers the synthesis of novel oligosaccharides, these results are highly encouraging as they uncover new features of GPs and start unfolding access to unique structures.

Although the synthetic potential of β-1,3-D-glucan phosphorylases towards long oligosaccharides remains underexplored compared to β-1,4-D-glucan phosphorylases, the biocatalytic synthesis of d-laminaribiose has been investigated in combination with other enzymes aiming at the industrial scale production in view of its biological importance and commercial applications. Many studies have demonstrated the outstanding in vitro activity of SCP as part of multi-enzyme synthetic processes in which downstream reactions catalysed by other phosphorylases use α-D-Glc-1P generated in situ through the conversion of expedient feedstock, thus leading to highly valuable sugars as already exemplified herein for β-1,4-linkage and broadly covered in recent reviews [23,133,134]. In this aspect, d-laminaribiose was produced from sucrose and D-Glc in a one-pot reaction containing individually immobilised SCP and *E. gracilis* extract with enriched LBP activity (Fig. 6C). The immobilised LBP onto Sepabeads retained over 50% activity when tested seven times, but the yield was low (20%). It has been suggested that the presence of other phosphorylases in the extract could generate longer oligosaccharides, which was confirmed by detection of laminaritriose [135]. The immobilised enzymes were also encapsulated with chitosan to improve thermal and operational stability. Significant increase of half-life (10-fold) and no activity loss after 12 times reuse were observed, but no yield improvement [136]. Additionally, the reportedly absence of an accessible recombinant LBP led the group to improve the LBP production in *E. gracilis* by testing different cultivation methods [137], although a recombinant preparation of LBP had been previously reported [124]. More recently, the overall improved immobilised two-enzyme system was used in a packed bed reactor for the first continuous production of d-laminaribiose. After optimisation of different parameters, the system exhibited operational stability during the course of 10 days and yielded over 46 g/L disaccharide maintaining the half-life of both biocatalysts [138]. Since the high production in continuous operation resulted in lower titre and purity, the system was further optimised by combining the adsorbent BEA zeolite in a consecutive packed bed column as a purification step. This approach increased the laminaribiose purity 200-fold, keeping a similar titre and yield [139].

Despite all the advantages of using sucrose as substrate and SCP as biocatalyst towards the synthesis of functional disaccharides, alternative multi-enzyme approaches have been also investigated using starch. Starch is cheaper than sucrose, the phosphorolysis produces more α-D-Glc-1P and the ratio α-D-Glc-1P/D-Glc can be tuned for optimisation [118]**.** In this sense, d-laminaribiose was successfully synthesised from starch and glucose by four thermophilic enzymes with 91% yield within 36 h. The process involved debranching of starch with isoamylase for conversion into α-D-Glc-1P by α-glucan phosphorylase and final catalysis by LBP (*Paenibacillus* sp YM-1) in the presence of external D-Glc. The maltose generated as by-product of α-glucan phosphorylase reaction was recycled into malto-oligosaccharides by 4-α-glucanotransferase for continuous production of α-D-Glc-1P (Fig. 6D). High concentrations of substrates were also tested to demonstrate the industrial potential of this system, but it became a limitation to be addressed [140]. The follow-up study involving starch as the only substrate and a mathematical model to predict optimal conditions led to high production of d-laminaribiose (>80%) from low and high concentrations of starch (Fig. 6B). Some data indicated that D-Glc was slowly released from starch and remained at low concentration, thus minimising not only inhibition of the enzymes but also the effect of Maillard reaction during the process at high temperature [118].

## 5 Conclusions

The use of natural glycosyltransferases and unnatural glycoside hydrolase mutants has broadened the scope for β-glucan synthesis, which, in tandem with phosphorylases, offers great diversity in producing designer β-glucans. The reconstitution of glycosyltransferases in vitro has enabled the polymerisation of glucose to form a variety of cellulose morphologies, whilst the advancements seen with glycosynthases offer great potential for the design of β-glucans with tailored functionalities. The current applications of β-1,4- and β-1,3-D-glucan phosphorylases highlight their relevance along with other important carbohydrate-active enzymes. From solo catalysis to multi-enzymatic cascades and from long-chain insoluble to short-chain soluble oligosaccharides, these wild-type enzymes are capable of producing a broad range of well-defined natural and unnatural structures in a controlled manner due to their tolerance to different reaction conditions and substrates. In particular, the oligomerisation-induced self-assembly of insoluble β-1,4 oligomers into highly ordered hierarchical nanostructures can be directed by simply tuning reaction conditions in the presence of natural substrates or using chemically functionalised substrates, thus representing a promising approach towards the bottom-up preparation of tailored cellulose-like materials. Considering what has been recently achieved, but also the prospect discovery of new phosphorylases [141], the great potential biocatalyst of such robust and versatile enzymes in academic research and industrial application becomes more evident.

## Declaration of competing interest

The authors declare that they have no known competing financial interests or personal relationships that could have appeared to influence the work reported in this paper.
